# Effectiveness of E-Self-help Interventions for Curbing Adult Problem Drinking: A Meta-analysis

**DOI:** 10.2196/jmir.1691

**Published:** 2011-06-30

**Authors:** Heleen Riper, Viola Spek, Brigitte Boon, Barbara Conijn, Jeannet Kramer, Katherina Martin-Abello, Filip Smit

**Affiliations:** ^5^Department of Epidemiology and BiostatisticsEMGO+ Institute for Health and HealthVU Unversity Medical CentreAmsterdamNetherlands; ^4^CoRPS Center of Research on Psychology in Somatic DiseasesTilburg UniversityTilburgNetherlands; ^3^Trimbos Institute, Netherlands Institute of Mental Health and AddictionUtrechtNetherlands; ^2^EMGO+ Institute for Health and Health Care ResearchVU University Medical CentreAmsterdamNetherlands; ^1^Department of Clinical PsychologyVU University AmsterdamAmsterdamNetherlands

**Keywords:** Meta-analysis, alcohol, problem drinking, randomized controlled trial, self-help, e-self-help, intervention, unguided self-help, low intensity interventions, Internet, adults

## Abstract

**Background:**

Self-help interventions without professional contact to curb adult problem drinking in the community are increasingly being delivered via the Internet.

**Objective:**

The objective of this meta-analysis was to assess the overall effectiveness of these eHealth interventions.

**Methods:**

In all, 9 randomized controlled trials (RCTs), all from high-income countries, with 9 comparison conditions and a total of 1553 participants, were identified, and their combined effectiveness in reducing alcohol consumption was evaluated by means of a meta-analysis.

**Results:**

An overall medium effect size (g = 0.44, 95% CI 0.17-0.71, random effect model) was found for the 9 studies, all of which compared no-contact interventions to control conditions. The medium effect was maintained (g = 0.39; 95% CI 0.23-0.57, random effect model) after exclusion of two outliers. Type of control group, treatment location, type of analysis, and sample size did not have differential impacts on treatment outcome. A significant difference (*P* = .04) emerged between single-session personalized normative feedback interventions (g = 0.27, 95% CI 0.11-0.43) and more extended e- self-help (g = 0.61, 95% CI 0.33-0.90).

**Conclusion:**

E-self-help interventions without professional contact are effective in curbing adult problem drinking in high-income countries. In view of the easy scalability and low dissemination costs of such interventions, we recommend exploration of whether these could broaden the scope of effective public health interventions in low- and middle-income countries as well.

## Introduction

The global economic and health burden of alcohol use disorders is widely recognized [1], as is the need for effective public health interventions to substantially reduce this burden [2,3]. Since the dawn of the new millennium, broad public access to the Internet and e-self-help has become a reality, and this has opened new avenues to reach out to the large, but relatively hidden, group of problem drinkers in the community [4]. Problem drinkers are defined as individuals who consume alcohol beyond the guideline for low-risk drinking. Different gradations of alcohol use disorders may underlie this excess in alcohol consumption. (See Table 1 and Textbox 1 for an overview of specified alcohol use disorders.) Ample meta-analyses have shown face-to-face screening and brief interventions (SBIs) to be effective [5], particularly in primary care [6] and college settings [7]. The wide-scale dissemination of SBIs in routine practice is hampered, however, by implementation barriers, including inadequate supporting policies and resources (time, money, and professional skills) in the health care sector [8] and by meager uptake by problem drinkers themselves [9]. E-self-help may provide a welcome extension to these SBIs. E-self-help interventions are available in both brief and more extended formats. *Single session e-personalized normative feedback* is a phrase used to describe a brief type of self-help delivered over the Internet. Personalized feedback refers to the provision of individualized observations on each drinker’s alcohol consumption patterns in comparison with the recommended low-risk drinking guidelines. Normative feedback is often an important component of these interventions, enabling problem drinkers to compare their own alcohol use (in terms of frequency, quantity, or other measures) to the level of their own cohort or peer group [10].

A more extended form of e-self-help consists of protocol-driven treatments based on principles of behavioral self-control [11], cognitive-behavioral therapy [12], motivational interviewing [13], or a combination of these. The recommended time of use of the extended self-help interventions is 6 weeks, as this is the expected time period in which changes in problematic alcohol use are appearing [14]. Potential benefits have already been illustrated in studies on e-self-help interventions that induce behavioral change in the use of substances such as alcohol or tobacco [15] or that treat mental health disorders like depression and anxiety [16-18].

The chief advantages of e-self-help interventions include their potential to reach broad groups of problem drinkers independent of time or geographical distance and at relatively low dissemination costs [19]. A recent review by Vernon and colleagues [20] has pinpointed similar reasons why users themselves find the interventions attractive, that is, they are timely, anonymous, accessible 24/7, and mostly free of charge. This is especially true of e-self-help interventions that participants can work through without involvement of a professional (defined here as *no-contact* interventions) that are offered for problem drinkers in the general population [21] or directed at students in college settings [22].

Studies investigating the effectiveness of e-self-help interventions among youth have been evaluated mostly in student settings in the United States and Australia and more recently in Europe [23-25]. In a meta-analysis conducted in 2009, Carey and colleagues [26] found a favorable impact of computer-based interventions on student alcohol consumption as compared with no-intervention controls. This favorable impact was also shown in a recent systematic review conducted in 2010 by White and colleagues [27] on the effectiveness of online programs for college and adult problem drinking. However, evaluation studies on e-self-help for student problem drinking in low- and middle-income countries (LMICs) are lacking.

Studies investigating the effectiveness of e-self-help interventions among adult problem drinkers are fewer in number, but they show promising results as well. Many can be characterized as feasibility studies with pretest-posttest designs [28], but the number of randomized controlled studies is on the rise [20]. The availability of evidence-based e-self-help interventions is growing in many high-income nations, including European countries [21,29,30], the United States [4], Canada [31], and Australia [32]. These countries have high Internet penetration rates and a strong public health focus on problem drinking.

We would argue for several reasons that e-self-help interventions could also benefit LMICs. First of all, the majority of people with alcohol use disorders in LMICs are not in treatment, and the many problem drinkers are not exposed to public health interventions at all because no appropriate strategies are in place [33]. The estimated treatment gap of 78% for people with alcohol use disorders in these countries serves to illustrate the many unmet needs [34]. Second, LMICs have meager health resources in terms of both finances and trained health professionals [33]. For countries with minimal resources and increasing problem drinking, such as India and China, low-cost e-self-help interventions might help to fill this public health gap [1,35]. Third, despite the promising results reported by a limited number of studies on face-to-face brief interventions in countries like Brazil [36], India [37], and Taiwan [38], LMICs still experience major obstacles to the full implementation of these SBIs, even more so than affluent countries [33,39]. Fourth, the high level of anonymity provided by self-help Internet interventions could be of value to problem drinkers in those LMICs, where face-to-face help for alcohol problems may be hampered by religious or cultural values that scorn alcohol use or professional help-seeking [40].

Before the scope of e-self-help interventions can be broadened in any type of country, their effectiveness needs to be evaluated beyond the individual studies that have been carried out so far on adult problem drinking. We have therefore conducted a meta-analysis of the currently available studies. As Web-based self-help interventions were preceded by CD-ROM interventions, we include studies on these as well. These CD-ROM studies used PC’s for the delivery of the intervention and applied recruitment strategies similar to the Web-based studies developed at a later stage. We hypothesized that e-self-help without professional guidance would prove effective in reducing problem drinking as compared with control groups that receive no interventions. Next, we examined whether a number of study characteristics impact the primary outcome measure of alcohol consumption. To the best of our knowledge, this paper is the first meta-analysis to report on the effectiveness of no contact e-self-help among adult problem drinkers.

**Alcohol use disorders**

Alcohol use disorders from the lexicon of alcohol and drug terms published by the World Health Organization [41]

- Abstinence is defined as refraining from drinking alcoholic beverages.
- Moderate drinking is the consumption of alcohol that does not exceed guidelines for moderate drinking in terms of volume or quantity per occasion.
- Heavy drinking is defined as drinking in excess of the standard of moderate drinking(see moderate drinking, above).
- Hazardous use (*Internation* *al Classification of Disease, Tenth R* *evision* [*ICD-10*] code Z72.1) is a pattern of heavy drinking and/or binge drinking that carries with it a risk of harmful consequences to the drinker. These consequences may be detrimental to physical or mental health, or have adverse social consequences to the drinker or others. Other potential consequences include worsening of existing medical conditions or psychiatric illnesses, injuries caused to self or others, due to impaired judgment after drinking, high risk sexual behavior while intoxicated, and worsening of personal or social interactions.
- Harmful drinking (*ICD-10* code F10.1) is a pattern of drinking that is causing damage to health. The damage may be either physical (eg, liver cirrhosis from chronic drinking) or mental (eg, depressive episodes secondary to drinking). Harmful patterns of use are often criticized by others and are sometimes associated with adverse social consequences of various kinds. Harmful drinking has persisted for at least 1 month or has occurred repeatedly over the past 12-month period; subject does not meet criteria for alcohol dependence.
- Alcohol dependence (*ICD-10* code F10.2) is defined as drinking that meets at least 3 of the following criteria: tolerance; withdrawal symptoms; impaired control; preoccupation with acquisition and/or use; persistent desire or unsuccessful efforts to quit; sustains social, occupational, or recreational disability; use continues despite adverse consequences.

## Methods

### Identification and Selection of Studies

**Figure 1:**
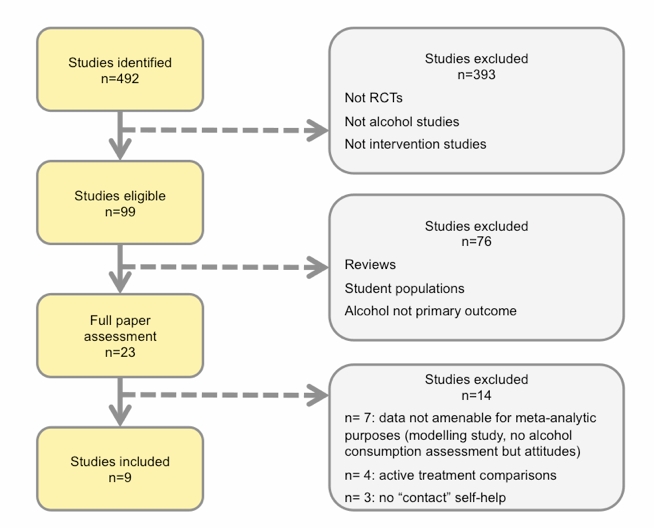
Flow chart of study selection resulting in inclusion of 9 studies and 9 comparisons

In February 2010 we carried out systematic searches of the literature in the following bibliographical databases: MEDLINE, PsycINFO (1997 to present), Science Citation Index Expanded, Social Sciences Citation Index, Arts and Humanities Citation Index (1997 to present), CINAHL, EMBASE, the Cochrane Drug and Alcohol Group Specialized Register, the Cochrane Effective Practice and Organization of Care (EPOC) Group register, the Alcohol and Alcohol Problems Science Database, and ETOH (etoh.niaaa.nih.gov, 1972 to 2003). Searches were conducted with keywords and text words, in which words indicative of eHealth interventions (Internet, Web, online, manual, and computer) were combined with terms indicative of alcohol disorders (alcohol abuse, alcoholism, problem drinking, hazardous drinking, harmful drinking, abstinence, moderation, treatment, brief intervention, self-help, and e-self-help) and our target group (adults). Those search strategies were combined with the optimal search strategy for randomized controlled trials (RCTs) designed by the United Kingdom Cochrane Centre (Cochrane Collaboration 2008). We also scanned Dissertation Abstracts and Digital Dissertations to cross-check references relating to earlier meta-analyses and systematic reviews on eHealth interventions, brief interventions, and e-self-help interventions for problem drinking as well as unpublished literature. Reference lists of retrieved papers were screened, and papers that possibly met inclusion criteria were retrieved and studied (Figure 1). No language restrictions were applied.

### Selection of Primary Studies

In this meta-analysis, we included only those studies that examined e-self-help interventions for adult problem drinkers (aged 18 or older). Studies on e-self-help interventions targeting student populations in college and university settings were excluded, as their effectiveness has been reported in other reviews and meta-analyses [22,26]. From studies that examined both problem and nonproblem drinkers (the latter not exceeding the guidelines for low-risk drinking), we entered the results for the problem drinkers only. We included only those randomized controlled trials that (1) compared e-self-help intervention groups with control groups, (2) reported data that were usable for meta-analytic procedures, and (3) assessed alcohol-drinking behavior (eg, frequency or quantity) as their primary outcome measure (Table 1).

Our initial selection from the first search was based on information derived from titles, abstracts, and keywords; if these yielded insufficient information to assess the inclusion criteria, then the full paper was retrieved. All papers excluded at this stage were independently assessed on their inclusion criteria by two independent raters (authors BB and HR) to ensure an error-free selection procedure. In addition, two independent coders, authors VS and HR, assessed the effect sizes and moderator variables of the included studies; any disagreement was resolved by discussion and consensus (Figure 1).

### Methodological Quality Assessment of Primary Studies

Some 25 scales are available to assess the validity and quality of RCTs [42]. As there is no evidence that more elaborate scales give more reliable assessments of validity than simpler ones, we chose an approach similar to that suggested by Higgins and Green [42] as well as approaches applied in two reviews of brief interventions for problem drinking in primary care [6,43]. This resulted in four basic criteria that we used to assess the validity and methodological quality of the studies we analyzed: (1) random allocation to condition performed by an independent third party, (2) random allocation concealed to study participants, (3) randomization status concealed to assessors of outcomes, and (4) completeness of follow-up data.

### Meta-analysis

We first examined the effects of e-self-help interventions for problematic alcohol use in comparison with control conditions. The usual approach is to calculate the standardized mean difference, also known as Cohen’s d (the mean difference divided by the pooled standard deviation) that is, d = (m_1_ – m_0_) / SD, where m_1_ and m_0_ are the mean scores in the experimental and control conditions.

The pooled standard deviation (SD) is defined as SD = √ ([n_1_–1]S_1_^2^ + [n_0_–1]S_0_^2^) / (n_1_ + n_0_ –2) where n_1_ and n_0_, and S_1_ and S_0_ are the sample sizes and variances of the experimental and control groups as obtained from the primary study. As this effect size d is subject to small-sample bias, it can be adjusted by using a scaling factor, which is multiplied by d to arrive at Hedges’ bias-corrected effect size g, where g = d (1– [3/4(n_1_ + n_0_] –9).

The interpretation of g is simple. An effect size of 0.5 indicates that the mean of the experimental group is half a standard deviation larger than the mean of the control group. In second-order meta-analysis, effect sizes of 0.56 to 1.20 may be interpreted as large, those from 0.33 to 0.55 as moderate, and those from 0 to 0.32 as small [44].

Our effect size calculations were performed on alcohol consumption measures only. If means and standard deviations were not reported, we used other statistics (*F* values and *P* values) to calculate effect sizes. If more than one measure was used in a single primary study, then the mean of the effect sizes was calculated so that each study was represented with only one effect size in our meta-analysis. In one study [45], two experimental conditions were compared with a control condition. In this case, the number of respondents in the control condition was divided equally over the two experimental conditions so that each respondent figured only once in the meta-analysis. In calculating pooled mean effect sizes, we used Comprehensive Meta-analysis (CMA) software, version 2.2.021 [46].

As we did not know before the analysis whether to expect heterogeneity among the studies, we used both the fixed effects model (FEM) and the random effects model (REM) to calculate pooled effect sizes. Heterogeneity was evaluated with the Q statistic and the I^2^ statistic. A significant Q rejects the null hypothesis of homogeneity, indicating that the variability among the effect sizes is greater than what would likely result from sampling error alone [47]. The I² statistic describes the percentage of total variation across studies, that is, attributable to systematic heterogeneity rather than chance alone. An I² value of 25% is associated with low heterogeneity, 50% with moderate, and 75% with high heterogeneity [48].

In subgroup analyses, we tested for significant differences between the effect sizes in different categories of studies using mixed effects analyses in CMA. We analyzed the following attributes: (1) type of treatment (single session personalized normative feedback versus extended self-help); (2) venue where intervention and assessments took place (research, health care, or workplace setting versus participants’ homes); (3) type of analysis (intention-to-treat versus completers only); (4) type of control condition (information, assessment-only, waiting list); and (5) small sample sizes (n < 100) versus large sample sizes (n > 100). Publication bias was tested by funnel plot and by Duval and Tweedie’s trim-and-fill procedure, which yields an estimate of effect size after publication bias has been taken into account (both procedures implemented in CMA).

### Description of Studies

A total of 492 studies were retrieved. Of these, 483 did not meet the inclusion criteria and were excluded (see Figure 1). A total of 9 trials with 9 comparisons and 1553 participants were ultimately included. Selected characteristics of those studies are summarized in Table 1. All 9 studies had been conducted in high-income countries: the United States (3), Canada (1), Netherlands (4), and Germany (1). Of these studies, 5 involved either single-session personalized normative feedback interventions while 4 involved more extended self-help interventions (see Table 1). 

**Table 1:** Selected characteristics of studies (N = 9)

Author, Year, Country	Mode of Delivery/ Setting^a^	Target Group, Inclusion Criteria	Intervention(s)/ Dose	Recruitment	N^b^	Control	Analysis and Timing of Posttreatment Assessment	Attrition Rate (%)
Boon and Huiberts, 2006, Netherlands [49]	Internet/ research setting	Males/females, ≥ 21/14 units/week and/or ≥ 6/4 units on ≥ 1 day/week	PNF^c^/ single session	Community	191	Alcohol leaflet	Completers only/ 9 months	32
Boon et al, 2011, Netherlands [50]	Internet/ research setting	Males, ≥ 21 units/week and/or ≥ 6 units on ≥1 day/week	PNF^c^/ single session	Community	450	Alcohol leaflet	Intention-to-treat/ 1 month	8
Cunningham et al, 2009, Canada [31]	Internet/ home	Alcohol Use Disorders Identification Test (AUDIT) ≥ 11	PNF^c^/ single session	Community	72	Alcohol leaflet	Intention-to-treat/ 3 months	8
Doumas and Hannah, 2008, United States [45]	Internet/ Work-place	Males/females, ≥1 occasions with ≥ 5/4 drinks in last 2 weeks	PNF^c^/ single session	Workplace	22	Assess-ment only	Completers only/ 1 month	37
Hester and Delaney, 1997, United States [51]	CD-ROM/ health care setting	AUDIT ≥ 8; males/females > 120/70 units/month or ≥ 6 units on ≥ 1 day/week	BSC^d^/ 8 sessions	Community	40	Waiting list	Completers only/ 10 weeks	0
Hester et al, 2005, United States [52]	CD-ROM/ health care setting	AUDIT ≥ 8	PNF^c^, BSC^d^, MI^e^/single session extened	Community	61	Waiting list	Completers only/ 4 weeks	0
Kramer et al, 2009, Netherlands [53]	TV/ Internet/ manual/ home	Males/females, > 21/14 units/week and/or ≥ 6/4 units on ≥ 1 day/week past month	BSC^d^, CBT^f^, MI^e^/ 5 sessions	Community	181	Waiting list	Intention-to-treat/ 5 weeks	6
Neumann et al, 2006, Germany [54]	Internet/ ED^g^	AUDIT ≥ 5	PNF^c^/ single session	Emergency department	275	Assess-ment only	Completers only/ 6 months	37
Riper et al, 2008, Netherlands [57]	Internet/ home	Males/females, >21/14 units/week or ≥6/4 units/≥1 day for past 3 months	BSC^d^, CBT^f^, MI^e^/ 6 weeks	Community	261	Alcohol leaflet	Intention-to-treat/ 6 months	42

^a^ At time of study; hence differences may exist between study and real life delivery

^b^ Number of problem drinkers

^c^ Personalized normative feedback

^d^ Behavioral self-control training

^e^ Motivational interviewing

^f^ Cognitive behavioral therapy

^g^ Emergency department

### Methodological Quality of Included Studies

All studies used well-validated alcohol consumption measures and well-described, theoretically based interventions [56]. In 4 studies [49,53,50,57], treatment was allocated by an independent third party; in 2 studies [31,45] it was not, and in 3 studies [51,52,54] this was unclear. Concealment of random allocation to participants was not applicable as study participants were recruited by self-reference and informed about the study conditions. In all but 3 studies [51,52,54] all outcome measures were self-administered by participants, making it irrelevant whether the researchers assessing the outcomes were blinded to group assignment. Dropout rates differed widely from 0% to 42% (Table 1).

## Results

**Figure 2:**
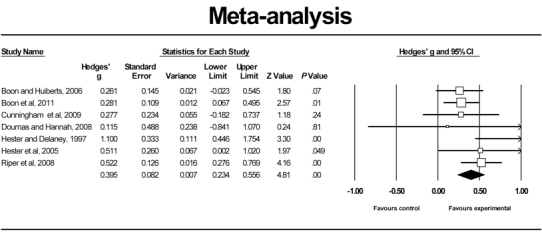
Meta-analysis of studies (omitting outliers)

Fixed effects meta-analysis of all studies resulted in a mean effect size of g = 0.39 (95% confidence interval [CI] 0.29-0.50). Random effects analysis resulted in a mean effect size of g = 0.44 (95% CI: 0.17-0.71). The hypothesis of homogeneity was rejected (Q = 42.30, I² = 81.08%, *P* < .001), thus lending preference to the random effects model. As possible sources of heterogeneity, we identified 2 outliers. The first was the Kramer study [53], with a large effect size of g = 1.11 (95% CI 0.80-1.42, random effects model). The main intervention component in this study consisted of five 25-minute televised sessions, a self-help manual, and a website. The second outlier was the Neumann study [54], with a small, negative effect size of g = −0.14 (95% CI −0.41 to 0.15, random effects model), which differed from other interventions in that recruited participants had just been treated at a hospital emergency department and were given an e-self-help intervention that did not address alcohol as such, but provided general lifestyle advice. Excluding these 2 outliers, we still obtained mean effect sizes of g = 0.39 (95% CI 0.23-0.56) in the random effects model (Q = 8.19, I² = 26.75%, *P* = .22) (see Figure 2). We also calculated the number needed to treat (NNT) as the effect size is not easy to interpret from a clinical point of view. We transformed the effect sizes based on *z* scores by using the formulae provided by Kraemer and Kupfer [58]. The results translate to an NNT of 5, indicating that about 5 problem drinkers must receive the intervention to generate 1 good treatment response. Posttreatment assessment was conducted at different points in time in different studies, ranging from 4 weeks to 9 months. Meta-regression analyses did not establish significant differences in intervention effects over time at posttreatment (beta = 0.00052, 95% CI = −0.00 to 0.009, *P* = .91).

### Subgroup Analysis

We then performed subgroup analyses to examine the contrast between extended e-self-help interventions and e-single-session personalized normative feedback interventions (omitting the outliers). Based upon the mixed effects model, these yielded a mean effect size of g = 0.27 (95% CI 0.11-0.43) for the e-single-session interventions and g = 0.61 (95% CI 0.33-0.90) for the extended e-self help interventions, a significant difference of *P* = .04 (Table 2). We found no significant differences between (1) where interventions and assessments were performed (research, health centre, or workplace versus places from where participants’ accessed the Internet, such as at home); (2) type of control condition (information, assessment-only, waiting-list); (3) small samples (n < 100) versus large samples (n > 100); nor (4) type of analysis (completers-only versus intention-to-treat).

### Sensitivity Analyses

The overall mean effect size was maintained even after exclusion of the largest study (N = 450) [50] in a random effects model (g = 0.44, 95% CI 0.24-0.64, Q = 6.87, I² *=* 27.23, *P* = .23). Duval and Tweedie’s trim-and-fill analysis did not detect publication bias (observed at g = 0.38, 95% CI 0.25-0.51, adjusted g = 0.35, 95% CI 0.23-0.47), nor did the funnel plot analysis detect bias. In view of these findings, we assume our post-treatment results to be robust up to 9 months [59].

**Table 2:** Effect sizes of e-interventions for problem drinking versus control conditions

Studies		Number of Comparisons	Hedges’ g	95% CI	*P*
All studies^a^		9	0.44	0.29-0.50	
All studies, outliers excluded^a^ [53,54]		7	0.39	0.23-0.57	
**Type of treatment**^b^					.04
	e-personalised normative feedback	4	0.27	0.11-0.43	
	e-self-help	3	0.61	0.33-0.90	
**Type of analysis**^b^					.60
	Intention-to-treat	3	0.37	0.21-0.54	
	Completers-only	4	0.48	0.11-0.86	
**Type of venue**^b^					.63
	Home	2	0.47	0.25-0.69	
	Research, health centre, or workplace setting	5	0.39	0.15-0.63	
**Sample size**^b^					.43
	Small	3	0.36	0.19-0.52	
	Large	4	0.52	0.14-0.91	
**Type of control condition**^b^					
	Alchol leaflet	4	0.35	0.21-0.48	.33
	Assessment only	1	0.12	−0.84 to 1.07	
	Waitlist control	2	0.77	0.19-1.34	

^a^ Random effect model

^b^ Mixed effects model

## Discussion

We found a medium effect size (g = 0.39) for eHealth interventions to reduce adult problem drinking in the general population up to 6 or 9 months posttreatment, as compared with no intervention. A significant difference (*P* = .04) emerged between e-single-session personalized normative feedback interventions (g = 0.27) and e-self-help interventions of a more extended nature (g = 0.61). This suggests that the latter may be more effective. Effects of the interventions beyond 9 months could not be assessed; one study reported 12-month follow-up results, and these suggested cost-effectiveness but a fade-out of the effect obtained at 6 months [55]; and one study was published after our search and found similar fade-out results at twelve months [60].

The medium effect size of our analysis compares favorably with the small effects reported for e-interventions in three recent meta-analyses. Rooke et al [15], Portnoy et al [61], and Riper et al [62] found small effect sizes for e-interventions for problem drinking. Some explanations may lie in differential characteristics of the interventions studied. The Riper study focused on single-session personalized normative feedback; the effect size of d = 0.23 was consistent with our present findings for this type of intervention (g = 0.27, 95% CI 0.11-0.42). Other explanations may involve characteristics of the control groups (active or nonactive). The study by Rooke et al, for example, included some active treatment comparisons, and these generally diminish effect sizes. The smaller effects in other meta-analyses may have also derived from the inclusion of both adult and college populations in some analyses, as e-self-help interventions are known to show smaller effects among younger age groups and student populations. This holds especially when these include prevention studies that include both alcohol drinking and nondrinking college students [26]. Of course, randomized controlled trials that compare different interventions in different populations are essential to assess the robustness of the various observations. The medium effect size in our analysis also compares well with effects reported for face-to-face adult brief interventions in primary care [6], for postal self-help interventions [63], and for brief interventions in non–treatment-seeking populations [5]. This further illustrates the potential of Internet interventions to extend the array of public health services to combat problem drinking.

### Limitations

Because our analysis is based on a rather limited number of studies, the results can be generalized only to self-referred adult problem drinkers in high-income countries recruited via the media; this implies samples of individuals with high readiness to change [64]. Second, some studies had small samples; we dealt with this by analyzing our data with Hedges’ g to adjust our estimates for small-sample bias [65]. Third, the loss to follow-up in some of the studies we reviewed was substantial. High dropout rates are a common feature of both online and offline self-help interventions for problem drinking and for Internet interventions in general [66,67]. Although some studies applied imputation techniques to handle the loss to follow-up, the high attrition may have biased our overall results.

### Implications for Clinical Practice and Future Research

The medium effect size we found for no-contact eHealth interventions could imply a major health impact at the population level, in view of the high percentages of problem drinkers that eHealth interventions might potentially reach. Naturally, eHealth interventions are subject to some constraints, as participants need computer and Internet access and a reasonable degree of literacy. While the costs of disseminating and scaling up no-contact eHealth interventions are low, the costs of developing them can be substantial [68].

Many questions still remain unanswered. We do not know yet whether e-self-help interventions that include professional contacts are more effective against problem drinking than no-contact interventions and, if so, to what *types* of problem drinkers that might apply (first-time help-seekers in the general population, primary- or secondary-care populations, hazardous or harmful drinkers, or dependent drinkers. (See Textbox 1.) This contrasts with e-self-help intervention studies focusing on common mental health disorders like depression and anxiety [69], which have established a firm evidence base for better clinical outcomes when e-self-help interventions are delivered with some form of professional guidance [18]. By contrast, Doumas and colleagues [45] have observed that a no-contact e-self-help intervention combined with 15 minutes of face-to-face motivational interviewing produced reductions in drinking that were comparable with the results of the no-contact e-self-help intervention alone. Nor could Rooke et al [15] establish any effect moderation attributable to personal guidance in alcohol e-interventions. As most participants in e-self-help interventions for problem drinking are first-time help-seekers [20,21], it could well be the case for this group that timely, anonymous access to e-interventions has a greater impact in altering drinking patterns than professional contact per se [70]. No-contact e-self-help interventions might therefore be an effective first-line choice in a stepped-care approach to problem drinking. A recently published study [71] on the posttreatment (3 months) effectiveness of intensive online treatment with active involvement of a therapist (duration of 3 months with 2 online therapist contacts a week) revealed an effect size of d = 1.21. This may indicate that therapist involvement will lead to higher effect sizes when compared with no-contact self-help interventions. More studies are needed, however, to assess the robustness of this observation and to assess how these more intensive treatments fit into a stepped-care approach.

From an economic point of view, no-contact e-self-help interventions could carry considerable promise as compared to other approaches like screening and brief face-to-face interventions in primary care, especially since the latter have relatively high implementation costs [72]. Studies that rigorously assess this proposition are not yet available [20], but economic evaluation studies on e-self-help for depression show favorable results as shown by a recent study of Gerhards and colleagues [73] in primary care and by Warmerdam and colleagues among depressed persons in the community [74]. The paper of Smit and colleagues in this issue shows that such economic advantages may also be expected from e-self-help intervention when implemented on a broad scale [75].

In light of all the potential benefits of e-self-help interventions in curbing problem drinking, we recommend their further evaluation and implementation as a means to bridging the gap in treating alcohol use disorders in low- and middle-income countries (LMICs). Although some may argue that this is not a viable option given the low Internet penetration in such countries, we would point out that such arguments abounded in high-income countries at the start of the millennium when e-self-help interventions for hazardous drinking were first being introduced. Meanwhile, the digital divide in Internet access between the more and less affluent countries is narrowing. In fact, one in four people worldwide now have Internet access [76]. Some e-self-help interventions have also proven well suited to be shared globally, as exemplified by the work of Munoz [77] on no-contact e-self-help for smoking cessation that are now used by people all over the world.

### Conclusion

Our study has sought to synthesize the available evidence about the effectiveness of no-contact e-self-help interventions in curbing adult problem drinking. The data suggest that these are effective. Our findings also highlight the need for more evaluations of the clinical outcomes and the cost-effectiveness of online screening instruments and interventions. The paper of Smit and colleagues in this issue indeed illustrates the economic benefits of e-self-help interventions for curbing adult problem drinking [75]. Future studies should shed light on whether e-self-help interventions produce similar or better results when extended with face-to-face components and on whether they could serve as alternatives or adjuncts to face-to-face treatments in primary care settings.
